# Takotsubo syndrome induced by *de novo* left bundle branch area pacing: a case report

**DOI:** 10.1093/ehjcr/ytae546

**Published:** 2024-11-05

**Authors:** Chokanan Thaitirarot, Shirley Sze, Ahmed Hafez, Raj Rajendra, Mokhtar Ibrahim

**Affiliations:** Department of Cardiology, Glenfield Hospital, University Hospitals of Leicester, Leicester LE3 9QP, UK; Cardiology Centre, Chulabhorn Hospital, Chulabhorn Royal Academy, Bangkok 10210, Thailand; Department of Cardiology, Glenfield Hospital, University Hospitals of Leicester, Leicester LE3 9QP, UK; Department of Cardiovascular Sciences, University of Leicester, Leicester LE1 7RH, UK; Department of Cardiology, Glenfield Hospital, University Hospitals of Leicester, Leicester LE3 9QP, UK; Department of Cardiology, Glenfield Hospital, University Hospitals of Leicester, Leicester LE3 9QP, UK; Department of Cardiology, Glenfield Hospital, University Hospitals of Leicester, Leicester LE3 9QP, UK

**Keywords:** Takotsubo syndrome, Conduction system pacing, Pacemaker, Complication, Case report

## Abstract

**Background:**

Takotsubo syndrome (TS), traditionally associated with emotional or physical stressors, manifests as transient left ventricular (LV) abnormalities mimicking acute coronary syndrome. Surgical procedures, such as pacemaker implantation, have emerged as potential TS triggers. Conduction system pacing (CSP), including His bundle pacing and left bundle branch area pacing (LBBAP), is a novel technique utilizing the heart's intrinsic conduction system. Despite documented cases of TS post-pacemaker implantation, the literature exploring the association between CSP and TS remains sparse.

**Case summary:**

In a case involving a 62-year-old woman with 2:1 atrioventricular block, an uncomplicated *de novo* LBBAP procedure was followed by post-procedural dizziness and dyspnoea. An initial transthoracic echocardiography revealed moderate–severe LV dysfunction, accompanied by elevated troponin levels. Coronary angiography showed unobstructed coronary arteries, while left ventriculography exhibited a classic apical ballooning. The patient had a favourable recovery, with LV function improvement noted before discharge.

**Discussion:**

Takotsubo syndrome may be triggered by other non-traditional physical stressors including traditional RV pacing and LBBAP. Clinicians should be aware of this potential, albeit rare, complication of LBBAP to ensure timely recognition and management. Increased awareness is vital for optimizing patient care during CSP procedures.

**Learning points**

- Conduction system pacing, including left bundle branch area pacing (LBBAP), is an emerging technique that aims to deliver a more physiological pattern of ventricular pacing compared with traditional right ventricular (RV) pacing.
- Takotsubo syndrome may be triggered by other non-traditional physical stressors including traditional RV pacing and LBBAP.
- Clinicians should be aware of this potential, albeit rare, complication of LBBAP to ensure timely recognition and management.

## Introduction

Takotsubo syndrome (TS), originally described by Japanese physicians in the 1990s, is a condition characterized by transient left ventricular (LV) wall motion abnormalities that closely mimic the presentation of acute coronary syndrome. While TS has traditionally been associated with emotional or physical stressors, it has become increasingly evident that surgical procedures, including pacemaker implantation, can serve as potential triggers for this condition.^1^ Conduction system pacing (CSP), encompassing His bundle pacing and left bundle branch area pacing (LBBAP), is an emerging approach to cardiac pacing. This novel technique utilizes the heart's intrinsic conduction system to enable efficient and physiologically synchronized ventricular activation.^2^ Despite documented cases of pacemaker implantation leading to TS, the literature on the association between CSP and TS remains limited. In this case report, we present a rare case of TS occurring after a successful *de novo* LBBAP procedure.

## Summary figure

**Figure:**
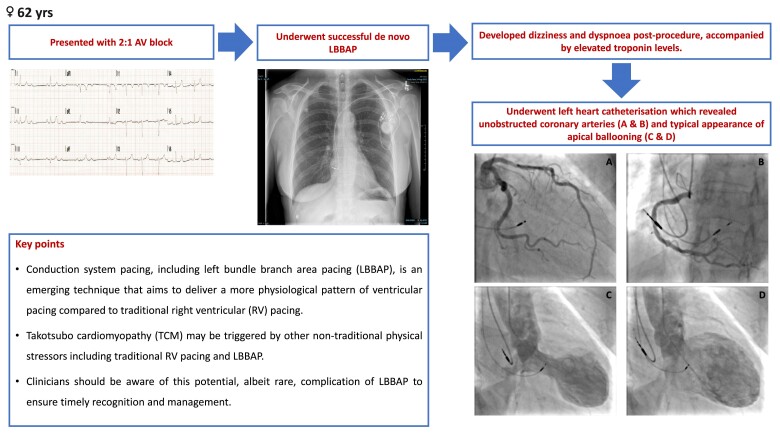


## Case report

**Figure 1:**
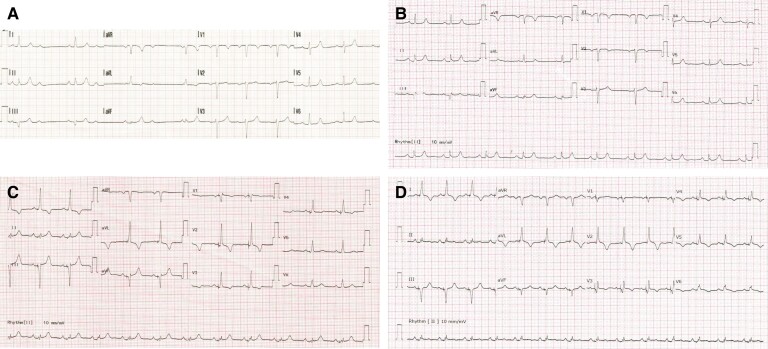
Electrocardiograms (ECG)s at different time points. (*A*) Pre-hospital ECG showed intermittent 2:1 atrioventricular block. (*B*) ECG at admission showed sinus rhythm with prolonged PR interval (206 ms). (*C*) Day 0 post-procedure ECG showed selective left bundle branch pacing. (*D*) Day 3 post-procedure ECG showed selective left bundle branch pacing with mild ST elevation and T wave inversion in V_2–6_.

**Figure 2:**
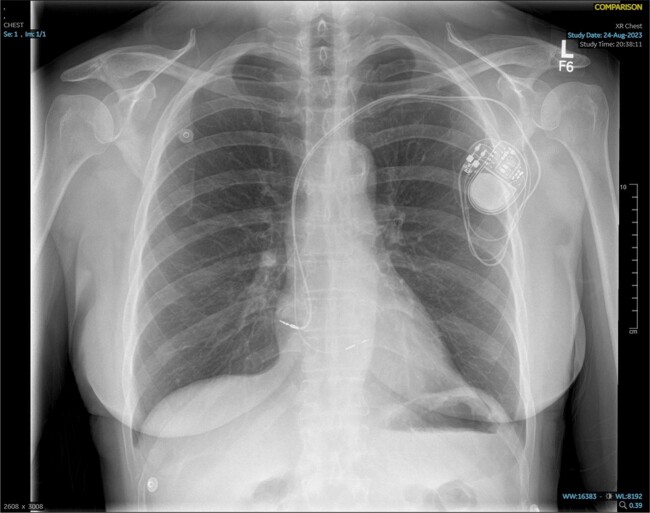
Chest X-ray (performed 2 h after left bundle branch area pacing) shows a dual-chamber permanent pacemaker with left bundle branch area pacing. A 69 cm SelectSecure lumenless 3830 lead was placed in the deep interventricular septum while a 52 cm CapSureFix Novus 4076 lead was positioned in the right atrial appendage.

A 62-year-old lady with history of posterior vitreous detachment and pulsatile tinnitus presented with palpitations and exertional dyspnoea. A pre-hospital 12-lead electrocardiogram (ECG) revealed intermittent 2:1 atrioventricular (AV) block while an admission ECG showed sinus rhythm with a prolonged PR interval (206 ms) (*Figure 1A* and *B*). A transthoracic echocardiography (TTE) showed normal LV size and systolic function [LV ejection fraction (LVEF) > 60%]. The patient subsequently underwent an uncomplicated *de novo* LBBAP procedure. A 69 cm SelectSecure lumenless 3830 lead was placed in the deep interventricular septum, while a 52 cm CapSureFix Novus 4076 lead was positioned in the right atrial appendage. These leads were connected to a magnetic resonance imaging (MRI)-compatible generator box (Azure DR MRI SureScan, Medtronic Inc.) (*Figure 2*).

Two hours after procedure, the patient developed dizziness, increasing shortness of breath, and chest tightness. Physical examination revealed tachypnoea and tachycardia, with blood pressure of 126/76 mmHg and oxygen saturation at 96% on room air. A 12-lead ECG during the event demonstrated atrial sensing and selective LBB capture without ST-T changes (*Figure 1C*). A bedside TTE ruled out pericardial effusion but revealed apical dyskinesia with moderate LV dysfunction (LVEF 35–40%) and mild mitral regurgitation. High-sensitivity troponin levels showed significant elevation [at 0 h 1335 ng/L and 3 h 4414 ng/L (Atellica IM High-Sensitivity Troponin I Assay)]. Pacing check was unremarkable, with atrial and ventricular pacing burdens of 5% and 97%, respectively.

**Figure 3:**
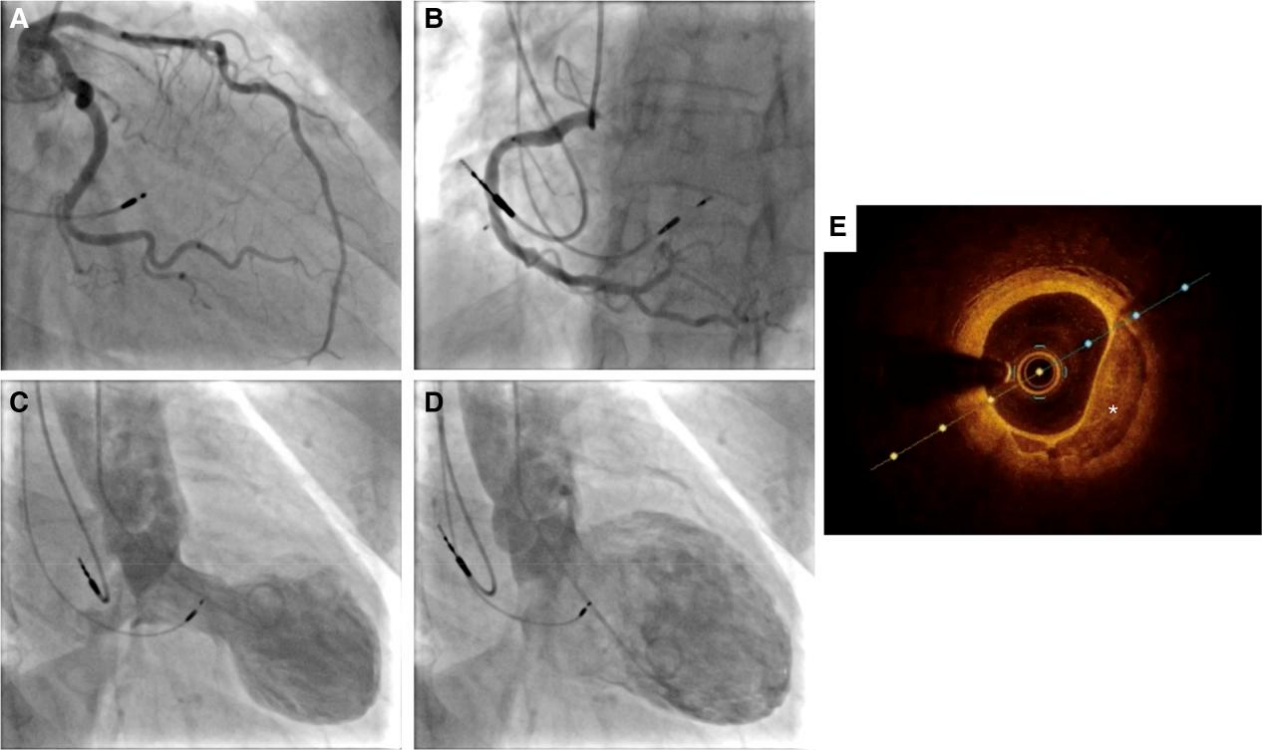
Coronary angiography, ventriculography and optical coherence tomography (OCT) image of proximal part of left anterior descending artery performed after left bundle branch area pacing. (*A*, *B*) Left and right coronary angiography, respectively, showing unobstructed coronary arteries. (*C*, *D*) Left ventriculography during systole and diastole, showing typical apical left ventricular ballooning and basal hyperkinesia. (*E*) OCT image of proximal part of left anterior descending artery showing eccentric fibrocalcific plaque with thin endocardial cap (marked with *).

Coronary angiography was promptly performed and showed a focal haziness in the proximal segment of the left anterior descending artery (*Figure 3A*). To further delineate this, an optical coherence tomography (OCT) study was undertaken and essentially showed eccentric fibrocalcific plaque with mild stenosis (<40%, minimal luminal area 3.94 mm^2^) with no evidence of plaque rupture or thrombus (*Figure 3E*). The rest of the coronary arteries were normal (*Figure 3A* and *B*). Left ventriculography displayed typical apical LV ballooning with LV end-diastolic pressure of 20 mmHg and no transaortic gradient (*Figure 3C* and *D*). On Day 3 post-procedure, a repeat 12-lead ECG showed mild ST elevation and T wave inversion in V_2–6_ (*Figure 1D*). A subsequent TTE on Day 5 post-procedure showed mildly impaired LV function (LVEF 45–50%). The patient was later discharged on Day 5 with ramipril 2.5 mg OD, bisoprolol 2.5 mg OD, and empagliflozin 10 mg OD. Videos of TTEs, coronary angiography, left ventriculography and OCT study can be found in Supplementary material.

## Discussion

We presented the case of a 62-year-old woman who developed TS following *de novo* LBBAP for 2:1 AV block. Left bundle branch area pacing was preferred to right ventricular (RV) pacing to provide more physiological ventricular activation, reducing the risk of pacing-induced cardiomyopathy in a patient expected to have a high ventricular pacing burden.

The exact aetiology of TS remains a subject of ongoing debate. Various contributing factors have been proposed such as catecholamine-induced myocardial stunning and inflammation, coronary microvascular dysfunction, and myocardial microinfarction.^3^ While TS is typically triggered by emotional or physiological stressors,^1^ there have been reports of TS being triggered by unconventional stressors such as lighting strike^4^ and hypoglycaemic attack.^5^ Surgical procedures, including pacemaker implantation, have also been implicated. In this case, there have been no emotional or physiological stressors before or during the hospital admission, with the exception of the LBBAP procedure.

Takotsubo syndrome typically utilizes imaging methods like ventriculography, echocardiography, and cardiac magnetic resonance (CMR) to ascertain the heart's structure and function.^6^ Specific CMR criteria for TS at the time of acute presentation have been established.^7^ With recent cardiac implantable electronic device implantation, some manufacturers advise against MRI until at least 6 weeks post-implantation, even for devices deemed MR conditional.^8^ Therefore, CMR was not performed.

A systematic review conducted by Strangio *et al*.^9^ evaluated the existing literature on pacemaker implantation-induced TS. This review included 28 patients from case reports. Women (75%) were predominantly affected with a mean age of 74 years. Most cases (93.2%) demonstrated full recovery of cardiac function, although the recovery period varied significantly, averaging around 7 weeks. It is important to note that all cases received first time pacemaker implantation. Notably, there has been only one previous case report of TS following LBBAP. Scuotto *et al*.^10^ described a case of a 93-year-old male who developed a much more severe form of TS following LBBAP. The patient underwent an extraction of a 14-year-old RV lead, followed by an implantation of a new LBB pacing lead into the deep interventricular position. After withdrawal of general anaesthesia, he developed cardiogenic shock requiring an intra-aortic balloon pump. It was possible that the extraction of the RV lead, rather than LBBAP, is a more likely culprit for TS. To our knowledge, this is the first case report of a patient who developed TS following uncomplicated *de novo* LBBAP.

Traditional RV pacing causes electrical and mechanical dyssynchrony, which is associated with an increased risk of atrial arrhythmias and heart failure.^11^ Conduction system pacing is an emerging technique that aims to deliver a more physiological pattern of ventricular pacing. It is steadily gaining interest and, with the availability of better delivery tools, is now an alternative to biventricular pacing for cardiac resynchronization therapy. His bundle pacing is limited by challenging implantation technique, high pacing capture threshold, and early battery depletion.^12^ In recent times, LBBP, defined as the capture of LBB via trans-ventricular septal approach, has emerged as a more promising alternative to address these issues.^13^

Awareness of potential complications associated with this novel technique is crucial. It is important to recognize TS as a potential, albeit rare, complication of LBBAP, that can have serious consequences. Nonetheless, the mainstay of treatment is largely supportive.

## Conclusion

In conclusion, we reported the first case report of TS following uncomplicated *de novo* LBBAP in a patient with 2:1 AV block. The case demonstrates the importance of considering TS as a potential complication of LBBAP and highlights the need for increased awareness during the perioperative management of patients undergoing CSP procedures.

## Lead author biography


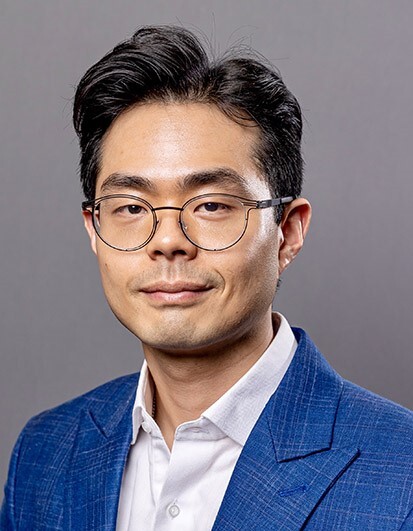
Dr Chokanan Thaitirarot is a dedicated cardiologist with subspecialty in heart failure and device therapy at Chulabhorn Hospital in Bangkok, Thailand. After obtaining a Certificate of Completion of Training (CCT UK), he pursued a clinical fellowship in Advanced Heart Failure, Mechanical Circulatory Support, and Heart Transplantation at Royal Papworth Hospital in Cambridge. He has strong interest in medical research and also has completed MSc in Evidence-Based Healthcare at University of Oxford.

## Supplementary Material

ytae546_Supplementary_Data

## Data Availability

Data are available upon reasonable request from the author at chokanan.thaitirarot@nhs.net.

## Supplementary material

**Consent:** The authors confirm that written consent for submission and publication of the clinical case including image(s) and associated text has been obtained from the patient in line with the COPE guidance.

**Funding:** None declared.
