# Supplementary material for: Takotsubo syndrome induced by de novo left bundle branch area pacing: a case report
Source: Eur Heart J Case Rep. 2024 Nov 5;8(11):ytae546. doi: 10.1093/ehjcr/ytae546 (PMC11538969; doi:10.1093/ehjcr/ytae546)
Supplement: ytae546_Supplementary_Data [file ytae546_supplementary_data.zip › Suppl mat for Takotsubo CM and LBBAP EHJ 130424.docx]

**SUPPLEMENTARY MATERIALS**

**Video 1** Transthoracic echocardiography two hours after left bundle branch area pacing (LBBAP) showing apical left ventricular (LV) ballooning with moderate-severe LV dysfunction (LV ejection fraction 35-40%), raising the initial suspicion of Takotsubo syndrome (TS)

**Video 2** Coronary angiography showing unobstructed coronary arteries

**Video 2.1** Left anterior oblique (LAO) caudal view of the left coronary artery

**Video 2.2** Posterior-anterior (PA) caudal view of the left coronary artery

**Video 2.3** Posterior-anterior (PA) cranial view of the left coronary artery

**Video 2.4** Right anterior oblique (RAO) caudal view of the left coronary artery

**Video 2.5** Right anterior oblique (RAO) cranial view of the left coronary artery

**Video 2.6** Left anterior oblique (LAO) view of the right coronary artery

**Video 2.7** Left anterior oblique (LAO) cranial view of the right coronary artery

**Video 2.8** Right anterior oblique (RAO) view of the right coronary artery

**Video 3** Left ventriculography displaying typical apical left ventricular (LV) ballooning

**Video 4** Optical coherence tomography of the left anterior descending (LAD) artery

**Video 5** Transthoracic echocardiography five days after left bundle branch area pacing (LBBAP) showing recovery of left ventricular (LV) function (LV ejection fraction 45-50%)

**Video 5.1** Apical 4-chamber view

**Video 5.2** Apical 2-chamber view
